# Comparative Effectiveness of Sodium-Glucose Co-transporter 2 (SGLT2) Inhibitors Versus Angiotensin Receptor-Neprilysin Inhibitors (ARNIs) in Heart Failure With Reduced Ejection Fraction: A Systematic Review

**DOI:** 10.7759/cureus.83166

**Published:** 2025-04-29

**Authors:** Prem Singh, Akhil Sunkara, Fnu Muskan, Kumari Varsha Lohana, Mahnoor Khan, Shadi S Al-Deir, Tajammul Abbas

**Affiliations:** 1 Neurology, Dow University of Health Sciences, Karachi, PAK; 2 Internal Medicine, Government Medical College Mahbubnagar, Mahabubnagar, IND; 3 Internal Medicine, Peoples University of Medical and Health Sciences for Women, Nawabshah, PAK; 4 Medicine and Surgery, Fazaia Medical College, Islamabad, PAK; 5 Internal Medicine, Misr University for Science and Technology, Amman, JOR; 6 Internal Medicine, Nishtar Medical University, Multan, PAK

**Keywords:** arni, cardiovascular outcomes, combination therapy, dapagliflozin, heart failure with reduced ejection fraction, hfref, randomized controlled trials, sacubitril/valsartan, sglt2 inhibitors, systematic review

## Abstract

Heart failure with reduced ejection fraction (HFrEF) remains a major cause of morbidity and mortality worldwide, despite advancements in pharmacotherapy. Among the most significant recent developments are sodium-glucose co-transporter 2 (SGLT2) inhibitors and angiotensin receptor-neprilysin inhibitors (ARNIs), both of which have demonstrated substantial improvements in clinical outcomes. This systematic review aimed to compare the efficacy, clinical outcomes, and therapeutic value of SGLT2 inhibitors versus ARNIs while also exploring their potential synergistic effects in the treatment of HFrEF. A comprehensive literature search was conducted across PubMed, Scopus, Embase, and Cochrane Central, adhering to Preferred Reporting Items for Systematic Reviews and Meta-Analyses (PRISMA) guidelines, and included randomized controlled trials published within the last 10 years. Five high-quality studies met the strict inclusion criteria, reflecting the limited but robust available evidence. The results suggest that both drug classes are effective in reducing cardiovascular death and heart failure hospitalizations, with emerging evidence indicating that their combined use may further enhance clinical outcomes. SGLT2 inhibitors have shown consistent benefits across key endpoints, even when used alongside ARNI therapy. The review highlights favorable safety profiles for both drug classes and supports early combination therapy in suitable patient populations. Observations regarding potential synergistic effects emerged from consistent trends across studies rather than being predefined primary outcomes. These findings reinforce current guideline recommendations advocating for multidrug strategies and emphasize the need for future direct comparative trials to optimize treatment sequencing in HFrEF.

## Introduction and background

Heart failure with reduced ejection fraction (HFrEF) remains a major public health burden, contributing to significant morbidity, mortality, and healthcare expenditure globally [1,2]. Despite advances in pharmacological and device-based therapies, the prognosis of patients with HFrEF has historically remained poor [3]. In recent years, two novel drug classes - angiotensin receptor-neprilysin inhibitors (ARNIs) and sodium-glucose co-transporter 2 (SGLT2) inhibitors - have emerged as pivotal disease-modifying agents, redefining the therapeutic landscape of HFrEF [4]. ARNIs, particularly sacubitril/valsartan, were introduced following the landmark Prospective Comparison of ARNI With Angiotensin-Converting Enzyme Inhibitor (ACEI) to Determine Impact on Global Mortality and Morbidity in Heart Failure (PARADIGM-HF) trial [5], which demonstrated substantial reductions in cardiovascular mortality and heart failure hospitalizations compared to enalapril. Subsequently, SGLT2 inhibitors, initially developed as antihyperglycemic agents, have shown compelling cardiovascular benefits independent of glycemic status, with large randomized controlled trials like Dapagliflozin and Prevention of Adverse Outcomes in Heart Failure (DAPA-HF) [6] and Empagliflozin Outcome Trial in Patients with Chronic Heart Failure and a Reduced Ejection Fraction (EMPEROR-Reduced) [7] highlighting their robust role in improving clinical outcomes in HFrEF patients.

Although previous systematic reviews have individually evaluated the efficacy of SGLT2 inhibitors or ARNIs, few have focused on their comparative effectiveness or potential additive benefits when used together. Given that both ARNIs and SGLT2 inhibitors are now considered foundational pillars of heart failure therapy, there is growing interest in understanding their comparative efficacy, safety profiles, and long-term outcomes [8]. While guidelines support the use of both drug classes - either sequentially or in combination - direct head-to-head comparisons are lacking, and clinicians are often faced with the challenge of optimizing sequencing and integrating these therapies in real-world practice. Our review addresses this gap by synthesizing comparative insights from high-quality trials, even in the absence of direct comparisons, to better inform clinical decision-making. Moreover, this review not only compares monotherapies but also explores the clinical impact of combining these therapies, particularly regarding cardiovascular outcomes, quality of life improvements, and adverse event profiles. These outcomes are of critical importance, as they directly influence patient prognosis, treatment adherence, and overall quality of care.

To guide the scope and structure of this systematic review, the Population, Intervention, Comparison, and Outcome (PICO) framework [9] was employed. The population (P) of interest includes adult patients diagnosed with HFrEF, generally defined as a left ventricular ejection fraction (LVEF) of 40% or less. The intervention (I) being evaluated comprises SGLT2 inhibitors, specifically dapagliflozin and empagliflozin, which have been studied extensively in the DAPA-HF and EMPEROR-Reduced trials, respectively. The comparator (C) is ARNI therapy, primarily sacubitril/valsartan, as investigated in the PARADIGM-HF trial and related follow-up analyses. The outcomes (O) of interest include changes in LVEF, all-cause mortality, cardiovascular death, heart failure-related hospitalizations, quality of life metrics, and adverse event profiles. This review, therefore, aims to systematically compare and interpret the evidence regarding the efficacy and safety of SGLT2 inhibitors versus ARNI therapy in improving these clinical outcomes among HFrEF patients, offering insights into which therapeutic strategy might offer superior or more sustainable benefits.

## Review

Materials and methods

Search Strategy

A comprehensive literature search was conducted following the Preferred Reporting Items for Systematic Reviews and Meta-Analyses (PRISMA) guidelines [10] to ensure methodological rigor and transparency. The search strategy was designed to identify relevant clinical trials comparing the efficacy and outcomes of sodium-glucose co-transporter 2 (SGLT2) inhibitors and ARNIs in patients with HFrEF. We systematically searched multiple databases, including PubMed, Scopus, Embase, and the Cochrane Central Register of Controlled Trials, using combinations of keywords such as "SGLT2 inhibitors", "ARNI", "heart failure", and "reduced ejection fraction". The search covered articles published from January 2014 to December 2023, aligning with the period when both drug classes became established in HFrEF management. Only randomized controlled trials and high-quality clinical trials published in English were included. Additionally, reference lists of included articles and major relevant reviews were manually screened to identify any missed studies. Grey literature sources, including clinical trial registries and conference abstracts, were not systematically searched. Observational studies, case reports, and review articles were excluded to maintain a focus on the highest level of clinical evidence relevant to our research objective.

Eligibility Criteria

The eligibility criteria for study inclusion were carefully defined to ensure relevance and quality of evidence for the comparison between SGLT2 inhibitors and ARNIs in the management of HFrEF. We included only randomized controlled trials or high-quality clinical trials that directly evaluated the efficacy, outcomes, or projected survival benefits of SGLT2 inhibitors (such as dapagliflozin or empagliflozin) and/or ARNIs (specifically sacubitril/valsartan) in adult patients with HFrEF, typically defined as LVEF of 40% or less. Eligible studies had to report at least one relevant clinical outcome such as cardiovascular death, heart failure hospitalization, functional capacity, or all-cause mortality. Subgroup analyses and exploratory modeling based on clinical trial data were also considered if they contributed meaningful insights into the comparative effectiveness or additive benefits of the two therapies.

Studies were excluded if they were observational in nature, case series, reviews, editorials, or non-English publications. Trials involving pediatric populations, patients with preserved ejection fraction, or those lacking clear outcome data specific to SGLT2 inhibitors or ARNIs were also excluded. In cases where multiple reports from the same trial were available, the most comprehensive or recent publication was selected. This strict inclusion and exclusion framework ensured that the final set of studies represented the most robust and clinically relevant evidence available for guiding the comparative assessment of these two major drug classes in HFrEF management.

Data Extraction

Data extraction was conducted independently by two reviewers using a standardized data collection form to ensure consistency and minimize bias. Prior to full data extraction, a calibration exercise was performed on a subset of studies to enhance inter-rater consistency and refine the data collection tool. For each included study, we extracted essential information such as author name, year of publication, study design, sample size, population characteristics, intervention details (SGLT2 inhibitor), comparator (ARNI or standard therapy), primary and secondary outcomes, and key findings. Additional data on subgroup analyses, baseline therapies, and outcome measures related to cardiovascular mortality, heart failure hospitalizations, and functional improvements were also recorded. Discrepancies between reviewers were resolved through discussion and consensus. If disagreement persisted, a third reviewer was available for adjudication to ensure data accuracy and completeness throughout the review process.

Data Analysis and Synthesis

Given the heterogeneity in study designs and the absence of direct head-to-head randomized trials comparing SGLT2 inhibitors and ARNIs, a qualitative synthesis approach was employed. Heterogeneity was assessed through visual inspection and systematic comparison of study designs, patient populations, background therapies, and outcome measures. The extracted data were systematically analyzed to compare the magnitude and consistency of treatment effects across studies, with particular attention to overlapping populations, baseline therapies, and shared clinical endpoints. A structured narrative synthesis was performed using thematic grouping and tabulation, organizing findings according to major outcome domains such as cardiovascular death, heart failure hospitalizations, functional improvements (e.g., peak VO₂), and safety profiles. Patterns, trends, and gaps in the literature were identified, and findings were narratively synthesized to provide a comprehensive comparative overview of the two drug classes in the context of HFrEF treatment.

Results

Study Selection Process

**Figure 1:**
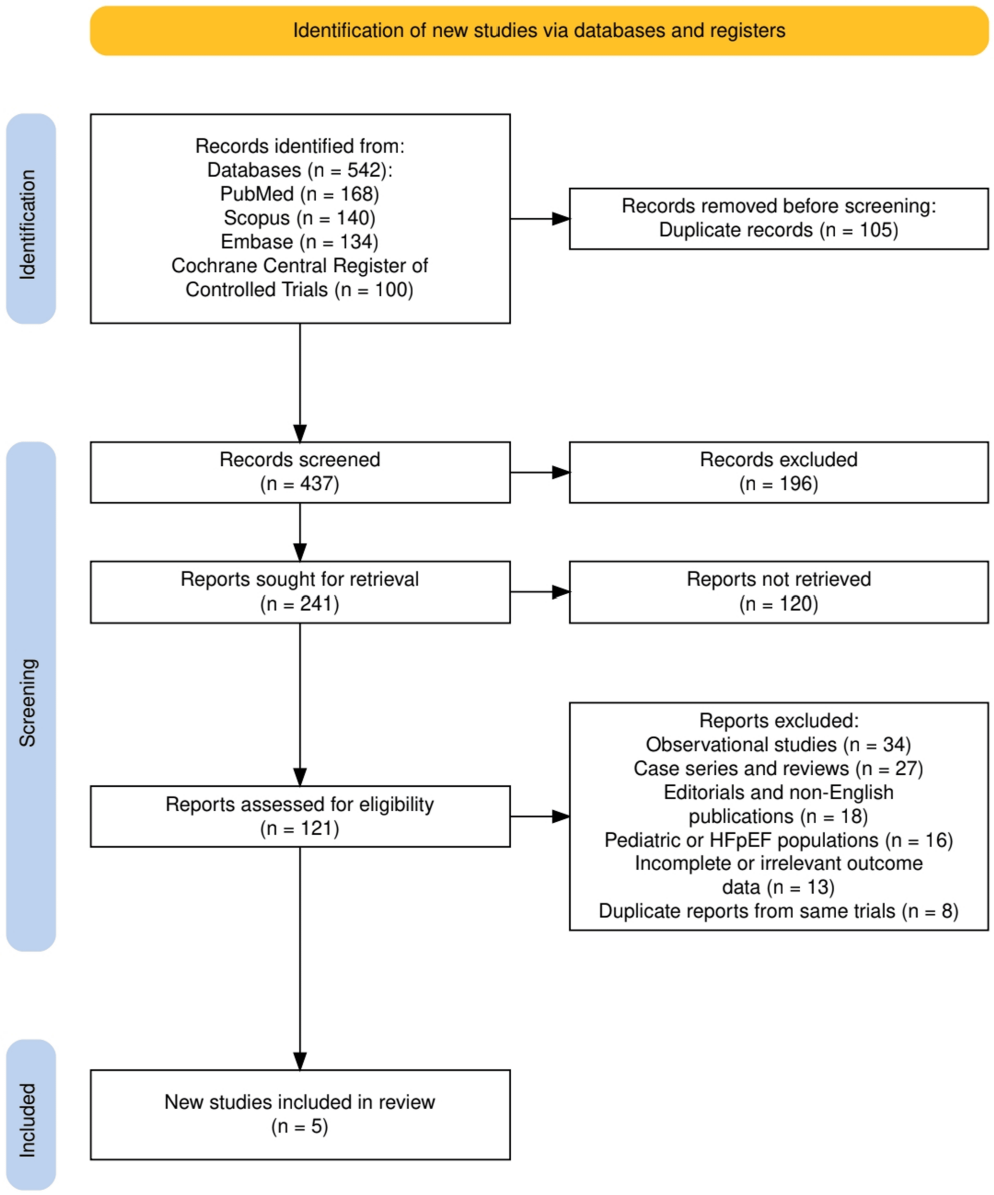
The PRISMA flowchart representing the study selection process. PRISMA: Preferred Reporting Items for Systematic Reviews and Meta-Analyses

The study selection process is illustrated in Figure 1, following the PRISMA flow diagram structure. A total of 542 records were identified through database searches, including 168 from PubMed, 140 from Scopus, 134 from Embase, and 100 from the Cochrane Central Register of Controlled Trials. After the removal of 105 duplicate records, 437 articles remained for screening. Following title and abstract screening, 196 records were excluded due to irrelevance to the review objectives. Of the 241 reports sought for retrieval, 120 were not retrievable, leaving 121 reports for full-text eligibility assessment. During this phase, 116 studies were excluded for reasons including being observational studies (n = 34), case series or reviews (n = 27), editorials or non-English publications (n = 18), focus on pediatric or preserved ejection fraction populations (n = 16), lack of relevant outcome data (n = 13), or duplicate reporting of the same trials (n = 8). Ultimately, five high-quality clinical trials met the eligibility criteria and were included in the final systematic review.

Characteristics of the Selected Studies

The characteristics of the studies included in this review are summarized in Table 1. All five selected studies were randomized controlled trials or analyses based on RCT data, focusing on patients with HFrEF. Sample sizes varied significantly, ranging from 90 participants in a functional capacity trial to over 13,000 patients assessed across multiple major heart failure trials. The study populations were broadly representative of real-world HFrEF cohorts, including individuals across a wide age range with varying degrees of comorbidities such as type 2 diabetes and renal impairment. All studies evaluated dapagliflozin as the SGLT2 inhibitor of interest, either alone or in conjunction with background therapy that included ARNIs. While some trials directly analyzed clinical outcomes such as cardiovascular mortality and heart failure hospitalizations, others focused on functional parameters or used modeling approaches to project long-term survival benefits. Importantly, most patients in these studies were already receiving optimized standard-of-care therapies, including β-blockers and mineralocorticoid receptor antagonists, which enhances the generalizability of the findings and provides valuable insight into the additive role of SGLT2 inhibitors and ARNIs in contemporary heart failure management.

**Table 1:** The characteristics of all of the selected studies. EMPHASIS-HF: Eplerenone in Mild Patients Hospitalization and Survival Study in Heart Failure; PARADIGM-HF: Prospective Comparison of ARNI with ACEI to Determine Impact on Global Mortality and Morbidity in Heart Failure; DAPA-HF: Dapagliflozin and Prevention of Adverse Outcomes in Heart Failure; DAPA-VO₂: Dapagliflozin and Peak Oxygen Uptake in Heart Failure; SGLT2i: sodium-glucose cotransporter-2 inhibitor; ARNI: angiotensin receptor-neprilysin inhibitor; ACEI: angiotensin-converting enzyme inhibitor; ARB: angiotensin receptor blocker; β-blocker: beta blocker; MRA: mineralocorticoid receptor antagonist; HFrEF: heart failure with reduced ejection fraction; EF: ejection fraction; BP: blood pressure; LVEF: left ventricular ejection fraction; NYHA: New York Heart Association; NT-proBNP: N-terminal pro B-type natriuretic peptide; T2DM: type 2 diabetes mellitus; QoL: quality of life; CV death: cardiovascular death; HF hospitalization: heart failure hospitalization; 6MWT: 6-minute walk test; RCT: randomized controlled trial; HR: hazard ratio

Study (author, year)	Study design	Sample size	Population characteristics	Intervention (SGLT2i)	Comparator (ARNI)	Primary outcomes	Key findings
Vaduganathan et al., 2020 [11]	Cross-trial comparative analysis of RCTs	EMPHASIS-HF (n = 2737), PARADIGM-HF (n = 8399), DAPA-HF (n = 4744)	Patients with chronic HFrEF; modeled across age groups (55-80 years); background of ACEI/ARB + β-blocker ± MRA	Dapagliflozin (from DAPA-HF)	Sacubitril/valsartan (from PARADIGM-HF)	Composite of CV death or first HF hospitalization; CV death; HF hospitalization; all-cause mortality	Comprehensive therapy including ARNI + SGLT2i + β-blocker + MRA showed a 62% relative risk reduction in the primary endpoint (HR 0.38), significant improvements in all individual outcomes, and extended event-free survival by up to 8.3 years compared to conventional therapy
Solomon et al., 2020 [12]	Subgroup analysis from RCT (DAPA-HF)	4744 total patients; 508 on sacubitril/valsartan at baseline	HFrEF patients with or without background ARNI; similar age, NYHA class, diabetes status; lower EF and BP in ARNI group	Dapagliflozin vs. placebo (with/without ARNI background)	Sacubitril/valsartan (background therapy for 10.7%)	CV death or worsening HF; all-cause mortality; safety outcomes	Dapagliflozin was equally effective in reducing CV death or HF worsening whether or not patients were on background sacubitril/valsartan (HR 0.75 vs. 0.74); no significant safety differences; results support additive benefit of combination therapy
McMurray et al., 2019 [13]	RCT baseline characterization (DAPA-HF)	4744	HFrEF (LVEF ≤ 40%, NYHA class ≥ II, elevated NT-proBNP); mean age 66; 23% women; 42% with known diabetes, 3% undiagnosed; 94% on ACEI/ARB/ARNI, 96% on β-blockers, 71% on MRAs	Dapagliflozin 10 mg daily (planned intervention)	Sacubitril/valsartan included in 94% ACEI/ARB/ARNI mix	Not applicable (baseline study); trial objective was to test dapagliflozin's added value on top of standard therapy	Patients in DAPA-HF were representative of contemporary HFrEF cohorts and were well-optimized on background therapy, with significant prevalence of diabetes and renal impairment—highlighting generalizability of the study population for SGLT2i evaluation
Palau et al., 2022 [14]	Double-blind, multicenter RCT (DAPA-VO₂)	90 (45 dapagliflozin, 45 placebo)	Stable HFrEF patients (mean age 67; 77% male); NYHA II; 29% with T2DM; high use of sacubitril/valsartan (88.9%), β-blockers (91.1%), and MRAs (74.4%)	Dapagliflozin 10 mg daily	Sacubitril/valsartan as background in 88.9%	Change in peak VO₂ at 1 and 3 months	Dapagliflozin significantly improved peak VO₂ at 1 month (+1.09 ml/kg/min) and 3 months (+1.06 ml/kg/min); no significant changes in 6MWT, QoL, or echocardiographic parameters; suggests early functional benefit of SGLT2i in well-treated HFrEF patients
Docherty et al., 2021 [15]	Exploratory extrapolation analysis from RCT (DAPA-HF)	4744	HFrEF patients (LVEF ≤ 40%, NYHA class II-IV); mean age 66.3; 23.4% female; global multicenter population; elevated NT-proBNP	Dapagliflozin 10 mg daily vs placebo	Not directly compared to ARNI, but conducted in patients on standard therapy including ARNI	CV death, HF hospitalization, urgent HF visits (composite); all-cause mortality	Extrapolated lifetime benefits showed dapagliflozin extended event-free survival by 2.1 years and overall survival by 1.7 years in a 65-year-old; consistent benefit seen across subgroups; supports long-term prognostic value of dapagliflozin in HFrEF

Quality Assessment

The quality assessment of the included studies is presented in Table 2, using the Cochrane Risk of Bias 2.0 (RoB 2) tool [16] to evaluate five key domains of methodological quality. Three studies were judged to have an overall low risk of bias, including the subgroup analysis, baseline characterization, and double-blind randomized controlled trial, all of which demonstrated clear randomization procedures, minimal deviations from intended interventions, complete outcome reporting, and objective outcome measurements. Two studies were rated as having some concerns, primarily due to the nature of their design. The cross-trial comparative analysis involved indirect comparisons between different studies, and the extrapolation study relied on model-based projections, both of which introduced uncertainty in terms of intervention fidelity and selective reporting. Despite these limitations, all five studies were deemed to be of high methodological quality overall and suitable for inclusion in this systematic review, given their rigorous design and relevance to the comparative evaluation of SGLT2 inhibitors and ARNIs in HFrEF.

**Table 2:** The quality assessment of all the included studies.

Study (author, year)	Study design	Randomization process	Deviations from intended interventions	Missing outcome data	Measurement of outcome	Selection of reported results	Overall risk of bias
Vaduganathan et al., 2020 [11]	Cross-trial comparison (based on RCTs)	Some concerns (indirect comparisons)	Some concerns (not a direct trial)	Low risk	Low risk	Some concerns	Some concerns
Solomon et al., 2020 [12]	Subgroup analysis of RCT	Low risk	Low risk	Low risk	Low risk	Low risk	Low risk
McMurray et al., 2019 [13]	Baseline characteristics study (DAPA-HF RCT)	Low risk	Low risk	Low risk	Low risk	Low risk	Low risk
Palau et al., 2022 [14]	Double-blind RCT	Low risk	Low risk	Low risk	Low risk	Low risk	Low risk
Docherty et al., 2021 [15]	Extrapolation from RCT data	Low risk	Some concerns (model-based extrapolation)	Low risk	Low risk	Some concerns	Some concerns

Discussion

The findings of this systematic review suggest that both SGLT2 inhibitors and ARNIs provide substantial clinical benefits in the management of HFrEF, with a potentially synergistic effect when used in combination. In the comparative analysis by Vaduganathan et al. [11], comprehensive disease-modifying therapy including both SGLT2 inhibitors and ARNIs, alongside beta-blockers and MRAs, resulted in a remarkable 62% relative risk reduction in cardiovascular death or first heart failure hospitalization, with up to 8.3 additional years of event-free survival in younger patients. The subgroup analysis from the DAPA-HF trial by Solomon et al. [12] reinforced these findings by demonstrating that dapagliflozin was equally effective in reducing cardiovascular events regardless of whether patients were on background sacubitril/valsartan, indicating that these agents can be safely and effectively co-administered. Additional support for the benefit of SGLT2 inhibitors was provided by Docherty et al. [15], who projected a gain of 2.1 years in event-free survival and 1.7 years in overall survival with dapagliflozin, further strengthening the case for its long-term use. Palau et al. [14] added evidence of early functional improvement, showing a significant increase in peak VO₂ within three months of dapagliflozin initiation in patients already receiving sacubitril/valsartan, though no major changes were noted in walk distance or quality of life. Importantly, McMurray et al. [13] confirmed the generalizability of the DAPA-HF trial population, which included a wide age range and high prevalence of diabetes, reflecting real-world HFrEF demographics. Taken together, these studies underscore the additive value of combining SGLT2 inhibitors and ARNIs, particularly in patients with advanced heart failure receiving optimized background therapy.

The results of this review have important implications for clinical practice, particularly in the context of evolving heart failure guidelines. Both SGLT2 inhibitors and ARNIs have demonstrated robust efficacy in reducing cardiovascular death, heart failure hospitalizations, and improving functional outcomes in patients with HFrEF [17]. Rather than being interchangeable, the evidence suggests that these drug classes are complementary, with additive benefits when used together. This aligns with recent recommendations from the American College of Cardiology/American Heart Association (ACC/AHA) and European Society of Cardiology (ESC) guidelines, which advocate for a multi-drug approach targeting different pathophysiological pathways [18]. Our review supports this therapeutic model by highlighting consistent benefits across studies when both SGLT2 inhibitors and ARNIs are incorporated into treatment regimens, reinforcing the trend toward early and comprehensive therapy in HFrEF management.

The clinical efficacy of both SGLT2 inhibitors and ARNIs can be attributed to their distinct and synergistic mechanisms of action. SGLT2 inhibitors, originally developed for glycemic control in diabetes, confer cardiovascular benefits through mechanisms such as osmotic diuresis, improved myocardial energy efficiency, reduction in preload and afterload, and renal protection by modulating glomerular hemodynamics [8]. In contrast, ARNIs act primarily through neurohormonal modulation by inhibiting neprilysin and blocking the angiotensin receptor, thereby enhancing natriuretic peptide activity while suppressing the deleterious effects of the renin-angiotensin-aldosterone system [19]. The combination of these mechanisms addresses different facets of heart failure pathophysiology, which may explain the observed additive or even synergistic effects, particularly in reducing hospitalizations and improving long-term outcomes.

Although the primary focus of this review was on efficacy, safety remains a critical consideration when implementing dual therapy. ARNIs are generally well tolerated but can be associated with hypotension, hyperkalemia, and rare cases of angioedema, particularly in patients with impaired renal function or low baseline blood pressure [20]. SGLT2 inhibitors, on the other hand, carry a risk of volume depletion, urinary tract and genital infections, and, in rare cases, euglycemic diabetic ketoacidosis [21]. However, the studies reviewed here did not reveal any significant increase in serious adverse events when these agents were used together, suggesting a favorable tolerability profile in well-selected patients. While individual tolerability should always guide therapy, the benefits appear to outweigh the risks, particularly when close monitoring is employed in clinical practice.

The findings of this review strongly support the concurrent use of SGLT2 inhibitors and ARNIs as part of a comprehensive treatment strategy for patients with HFrEF, rather than viewing them as sequential or competing options [22]. Evidence from subgroup analyses, particularly the DAPA-HF trial, indicates that the benefits of dapagliflozin are preserved regardless of background sacubitril/valsartan therapy, suggesting that SGLT2 inhibitors retain their efficacy even when added to optimized ARNI-based regimens. This supports a flexible, patient-centered approach to treatment sequencing [23]. In clinical practice, SGLT2 inhibitors may be particularly advantageous as a first-line addition in patients with lower blood pressure or renal impairment, where ARNIs might pose a higher risk of hypotension or hyperkalemia. Conversely, ARNIs may be prioritized in patients with more pronounced neurohormonal activation or those transitioning from ACE inhibitors. Overall, the data advocate for early integration of both drug classes, tailored to individual patient profiles, to maximize therapeutic benefit and improve long-term outcomes. While the combined use of SGLT2 inhibitors and ARNIs shows promising efficacy, treatment decisions should be individualized based on patient-specific factors such as comorbidities, tolerability, and socioeconomic considerations, recognizing that unanswered questions remain regarding optimal sequencing, long-term safety, and accessibility across different healthcare settings.

This review is strengthened by its exclusive reliance on recent, high-quality randomized controlled trials, ensuring that the evidence synthesized is both current and clinically relevant. The methodology adhered to PRISMA guidelines and employed the Cochrane RoB 2 tool for quality assessment, enhancing the transparency and rigor of the analysis. By focusing on SGLT2 inhibitors and ARNIs - the two most transformative drug classes in modern HFrEF management - the review provides a clear, focused comparison of their efficacy and potential synergy. However, certain limitations must be acknowledged. The included studies varied in design, encompassing modeling and extrapolation analyses, randomized controlled trial subgroup analyses, and functional capacity trials, which introduce clinical heterogeneity. The reliance on indirect comparisons, particularly in modeled analyses like those by Vaduganathan et al. [11], further limits definitive comparative conclusions. Differences in outcome measures, such as functional endpoints (e.g., peak VO₂) versus hard clinical outcomes (e.g., mortality and hospitalization), also complicate direct comparisons across studies. Additionally, while the overall generalizability of findings is strong, demographic gaps remain, particularly the underrepresentation of older adults (>80 years), women, and ethnic minorities, which warrants cautious interpretation. Moreover, the available data on long-term safety outcomes and subgroup responses in patients with comorbidities such as advanced renal dysfunction or diabetes remain limited, emphasizing the need for further research to guide personalized therapy.

Future research should prioritize the execution of direct head-to-head randomized controlled trials comparing SGLT2 inhibitors and ARNIs to definitively establish their relative efficacy, particularly in diverse patient populations. While current evidence supports their combined use, understanding which therapy may offer superior benefit in specific clinical contexts remains an open question. Additionally, biomarker-driven approaches - such as utilizing natriuretic peptides, renal function markers, or inflammatory profiles - could refine patient selection and guide personalized therapy strategies [24,25]. Further investigation into the cost-effectiveness of these therapies, as well as factors influencing patient adherence and real-world tolerability, will be essential for optimizing long-term outcomes and resource utilization in heart failure management.

## Conclusions

This review reinforces the essential role of both SGLT2 inhibitors and ARNIs in the modern management of HFrEF. Rather than being alternatives, these therapies offer complementary mechanisms and additive clinical benefits when used together, supporting their early and combined use in appropriate patients. The consistent improvements in cardiovascular outcomes, functional capacity, and projected survival across studies highlight the importance of integrating both drug classes into guideline-directed medical therapy. This review positions the combination of SGLT2 inhibitors and ARNIs as a cornerstone of contemporary HFrEF treatment, signaling a shift toward a more aggressive and multidimensional pharmacologic strategy aimed at improving long-term patient outcomes.
